# Mutational screening of inverted formin 2 in adult-onset focal segmental glomerulosclerosis or minimal change patients from the Czech Republic

**DOI:** 10.1186/s12881-018-0667-9

**Published:** 2018-08-20

**Authors:** Marketa Safarikova, Jitka Stekrova, Eva Honsova, Vera Horinova, Vladimir Tesar, Jana Reiterova

**Affiliations:** 1 Institute of Biology and Medical Genetics, First Faculty of Medicine, Charles University and General University Hospital in Prague, Prague, Czech Republic; 2 Department of Nephrology, First Faculty of Medicine, Charles University and General University Hospital in Prague, Prague, Czech Republic; 3 Department of Clinical and Transplant Pathology, Institute for Clinical and Experimental Medicine, Prague, Czech Republic; 4 Reprofit International, Brno, Czech Republic

**Keywords:** Focal segmental glomerulosclerosis, Minimal change disease, End stage renal disease, High resolution melting method, *INF2*

## Abstract

**Background:**

Mutations in *INF2* are frequently responsible for focal segmental glomerulosclerosis (FSGS), which is a common cause of end stage renal disease (ESRD); additionally, they are also connected with Charcot-Marie-Tooth neuropathy. *INF2* encodes for inverted formin 2. This protein participates in regulation of the dynamics of the actin cytoskeleton, involving not only the polymerisation, but also the depolymerisation of filaments. The present study is the first mutational analysis of *INF2* done in the Czech Republic.

**Methods:**

Mutational analysis of *INF2* was performed on 109 patients (mean age at onset 41.44 ± 18.91 years) with FSGS or minimal change disease (MCD); and also in 6 patients without renal biopsy who had already developed chronic kidney disease (CKD)/ESRD at the time of diagnosis. We used high resolution melting method (HRM), with subsequent Sanger sequencing, in suspect samples from HRM analysis. The HRM method is an effective method for the screening of large cohorts of patients.

**Results:**

Two pathogenic mutations (p.Arg214His and p.Arg218Gln) were detected in *INF2*. The first (p.Arg214His) was identified in the FSGS patient with a positive family history. The second mutation (p.Arg218Gln) was found in two brothers with ESRD of unknown etiology. The most frequent sequence change was the substitution p.P35P, the incidence of which corresponded with the frequencies available in the ExAC Browser and gnomAD Browser databases. This analysis also detected different exonic and intronic changes that probably did not influence the phenotype of the included patients.

**Conclusions:**

The *INF2* mutational screening is useful in familial FSGS cases as well as in patients with an unknown cause for their ESRD, but with a positive family history. *INF2* seems to be not only the cause of FSGS, but also of ESRD of unknown etiology. Our study has confirmed that the HRM analysis is a very useful method for the identification of single nucleotide substitutions.

## Background

Podocytes are highly specialized cells with a unique and highly differentiated cytoarchitecture. Their main functions include: support of the glomerular capillaries, synthesis of glomerular basement membrane (GBM), and regulation of permselectivity. Any damage to the podocytes is usually characterized by nephrotic syndrome and (if persistent) it may progress into end stage renal disease (ESRD). Minimal change disease (MCD) and focal segmental glomerulosclerosis (FSGS) are common causes of nephrotic syndrome.

The major pathological feature of both MCD and FSGS is increased permeability of the glomerular capillary filter. Under light microscopy the glomeruli of patients with MCD have a normal size and cellularity and show no evidence of segmental sclerosis; whereas electron microscopy demonstrates the same podocyte foot processes effacement as in glomeruli affected by FSGS. Whereas the outcome of MCD in terms of long-term preservation of renal function is good; by comparison, FSGS often leads to ESRD. We still have only limited insight into the pathogenesis of both MCD and FSGS. Moreover, both the etiology and histology of FSGS as well as of MCD may be very variable, including: viral infections, toxic agents, adaptive structural-functional responses, and mutations in the genes encoding proteins specific for podocytes (or highly expressed in them) [1–3], such as: *ACTN4* [4]*, INF2* [5], and *TRPC6* [6].

Inverted formin 2, encoded by *INF2*, is a member of the formin family of proteins, which regulates the dynamics of the actin cytoskeleton. It not only accelerates actin polymerisation, but also filament depolymerisation. INF2 is expressed in many tissues such as: kidney, liver, heart, skeletal muscles, and placenta. In renal podocytes, it plays a key role in the function and structure of the actin cytoskeleton. The main regions of INF2 are: the N-terminal diaphanous inhibitory domain (DID), the formin homology FH1 and FH2 domains, and the terminal diaphanous autoregulatory domain (DAD) [7, 8]. Most of the reported mutations are localized in the DID domain, which probably functions as a regulatory domain of the polymerisation and depolymerisation of the actin filaments. The previous experiments demonstrated that substitutions in INF2 caused an abnormal distribution of the slit diaphragm proteins nephrin and podocin, and dysregulation of the podocyte cytoskeleton; suggesting its involvement in the pathogenesis of the autosomal dominant form of FSGS [5, 9]. Mutations in *INF2* are not only connected with FSGS, but also with Charcot-Marie-Tooth neuropathy [10].

In this study, we present a mutational analysis of *INF2* (whose mutations are frequent causes of FSGS in adults) in patients with FSGS or MCD as well as in a group of patients with a positive family history of ESRD of unknown etiology, using a high resolution melting method (HRM) and Sanger sequencing.

## Methods

### Patients and study design

Our study, focused upon *INF2*, was carried out in 109 patients with biopsy-proven FSGS or MCD (see Table 1 for detailed information). The origins of FSGS or MCD of the involved individuals were both familial (inherited) in 19 cases, and idiopathic (sporadic). The study also included a group of 6 patients with a positive family history for ESRD in combination with developed advanced chronic kidney disease (CKD)/ESRD at the time of diagnosis. There were no renal biopsies performed in this group of patients thought to have burnt out diagnostic features because of advanced disease. Healthy controls from anonymous volunteers were used to test for variants in healthy individuals.

**Table 1:** Clinical data of 109 patients with FSGS/MCD included in the analysis of *INF2*

Characteristic	Group	Number/Value
Diagnosis	FSGS	77 (70.6%)
	MCD	32 (29.4%)
Sex	Male	51 (46.8%)
	Female	58 (53.2%)
Family history	Positive	19 (17.4%)
	Negative	90 (82.6%)
NS at the time of diagnosis	Yes	78 (71.6%)
	No	31 (28.4%)
Effect of corticosteroid therapy	Sensitive	34 (31.2%)
	Resistant	41 (37.6%)
	No therapy	34 (31.2%)
Mean age at the time of diagnosis	Years	41.44 ± 18.91
Mean proteinuria at the time of diagnosis	All: Grams/24 h	5.67 ± 4.64
	FSGS: Grams/24 h	4.99 ± 4.14
	MCD: Grams/24 h	7.42 ± 5.32
Mean serum albumin level at the time of diagnosis	All: Grams/Litre	28.05 ± 9.48
	FSGS: Grams/Litre	30.36 ± 9.1
	MCD: Grams/Litre	22.15 ± 7.67
Average serum creatinine level at the time of diagnosis	All: μmol/Litre	109.52 ± 61.26
	FSGS: μmol/Litre	112.05 ± 52.21
	MCD: μmol/Litre	103.31 ± 78.91

Data are presented by mean ± SD or number (percentage)

*FSGS* Focal segmental glomerulosclerosis, *MCD* Minimal change disease, *NS* Nephrotic syndrome (defined as presence of proteinuria ≥3 g/24 h plus serum albumin level ≤ 30 g/L)

### Ethical approval

The study was performed with the approval of the Ethics Committee of the General University Hospital in Prague. The blood of patients, their relatives, and the anonymized controls was obtained after written informed consent was given by all in accordance with the protocol approved by the institutional review board at the General University Hospital.

### Mutational analysis

Genomic DNA was isolated from the peripheral blood lymphocytes using standard procedures. The complete coding region and intron-exon boundaries of *INF2* were amplified, and were subsequently analyzed using the HRM method and LightCycler 480, as well as HRM Master Mix (both by Roche Diagnostics). The data obtained from the HRM analysis were analyzed using Light Cycler Gene Scanning Software (Roche Diagnostics). The suspect samples from the HRM analysis were screened for substitutions by Sanger sequencing performed on an ABI Prism™ 3130 Genetic Analyser using BigDye® Terminator v1.1 Cycle Sequencing Kit (both by Applied Biosystems). All primer sequences and annealing temperatures are available upon request. Targeted Sanger sequencing was also performed in the parents and relatives of probands with causal mutations or novel substitutions in order to determine their carryover and position on homologous chromosomes.

The HRM method is a fast, simple, and cheap method for the analysis of large cohorts of patients. Especially for SNP scanning, this is a highly sensitive and specific method, with some limitations (e.g., GC-rich fragments, or detection of homozygous variants). Sanger sequencing is the technique commonly used in molecular laboratories. On the one hand, it is a simple method in terms of sample preparation, and an effective way to analyze single genes, or to confirm variations identified by next generation sequencing. Then again, the procedure is an expensive procedure with detection limitations (e.g., mosaicism).

### Sequence analysis and assessment of sequence changes

Sequence chromatograms were analyzed using a BioEdit Sequence Alignment Editor (http://www.mbio.ncsu.edu/BioEdit). All sequenced samples were compared to the reference coding sequence NM_022489.3 as well as the reference genome sequence NG_027684.1, respectively. Substitutions found in this analysis were checked against the Human Gene Mutation Database (http://www.hgmd.cf.ac.uk/ac/index.php) and/or the Ensembl [11] and NCBI dbSNP database (https://www.ncbi.nlm.nih.gov/projects/SNP/). In the case of novel missense variants, their pathogenicity was assessed computationally using Mutation Taster [12], PolyPhen-2 [13], PROVEAN [14], and SIFT [15]. The possible splice effect of intronic changes was evaluated using Human Splicing Finder [16] and NetGene2 (http://www.cbs.dtu.dk/services/NetGene2/).

## Results

*INF2* mutational analysis was performed in a total of the 115 patients with FSGS (77 patients) /MCD (32 patients), or the characteristics of a separate group including a positive family history for ESRD in combination with developed advanced CKD/ESRD at the time of diagnosis. The described mutations and both the exonic and intronic polymorphisms had been identified. See Table 2 for the exonic variants identified.

**Table 2:** All *INF2* exonic variants found in our two cohorts of patients including allele frequencies from ExAC Browser and gnomAD Browser

Exone	cDNA level	Protein level	Allele frequency % (*n* = 115)	ExAC Browser Af	gnomAD Browser Af	Reference or rs number
2	c.42 G > A	p.Leu14Leu	0.9	0.03997	0.01817	rs62638758
2	c.105 C > T	p.Pro35Pro	97.4	0.9783	0.9783	rs4983530
4	c.579 C > T	p.Tyr193Tyr	0.4			Novel
**4**	**c.641 G > A**	**p.Arg214His**	**0.4**	**NF**	**NF**	**[5]**
**4**	**c.653 G > A**	**p.Arg218Gln**	**0.9**	**NF**	**NF**	**[5]**
7	c.879G > A	p.Ser293Ser	0.9	0.007779	0.007394	rs184709736
7	c.885G > A	p.Leu295Leu	0.4	0.00009769	0.00009.272	rs370680236
8	c.1472C > T	p.Pro491Leu	0.4			Novel
8	c.1499C > T	p.Pro500Leu	0.4	0.0009303	0.0003025	rs561201601
8	c.1582C > T	p.Pro528Ser	0.9	0.007560	0.005266	rs181694819
18	c.2640 T > C	p.Asp880Asp	60.0	0.8608	0.8619	rs10133301
21	c.3066 T > C	p.Asp1022Asp	68.3	0.8060	0.7871	rs4983535
21	c.3108 T > C	p.Leu1036Leu	0.4	0.007002	0.004700	rs186075307
21	c.3163C > T	p.Pro1055Ser	0.4			Novel
21	c.3169C > T	p.Pro1057Ser	2.2			Novel
21	c.3170C > T	p.Pro1057Leu	1.3			Novel
21	c.3177C > T	p.Pro1059Pro	0.9			Novel
21	c.3179C > T	p.Thr1060Ile	3.5			Novel
21	c.3180C > T	p.Thr1060Thr	1.7			Novel
21	c.3181C > A	p.Leu1061Met	0.9			Novel
21	c.3181C > T	p.Leu1061Leu	1.3			Novel
21	c.3207A > C	p.Pro1069Pro	40.4	0.6103	0.6053	rs1128840
21	c.3207A > G	p.Pro1069Pro	40.9	0.2469	0.2524	rs1128840
21	c.3286C > T	p.Pro1096Ser	7.8	0.07527	0.06494	rs34251364

Pathogenic mutations are showed in bold. Allele frequency was counted for the current study

*Af* Allele frequency

*NF* Not found in browser

Two mutations in *INF2* (p.Arg218Gln and p.Arg214His) already known to be responsible for the familial form of FSGS [5] were detected in two different families with a positive family history for FSGS, or ESRD of unknown etiology. Both of the mutations found are localized in exon 4, which is involved in the highly conserved DID region, and that is situated at the N-terminus of the INF2 protein.

The most frequent substitution in *INF2* (p.P35P) located in exon 2, had been found in all patients, mostly in the homozygous state (allele frequency = 97.4%). Exon 21 of *INF2* seemed to be a very variable region because of the high number of changes identified. However, these substitutions probably did not influence the phenotype of the patients included in our study.

### Family no. 133 (*INF2*, p.Arg218Gln)

**Fig. 1:**
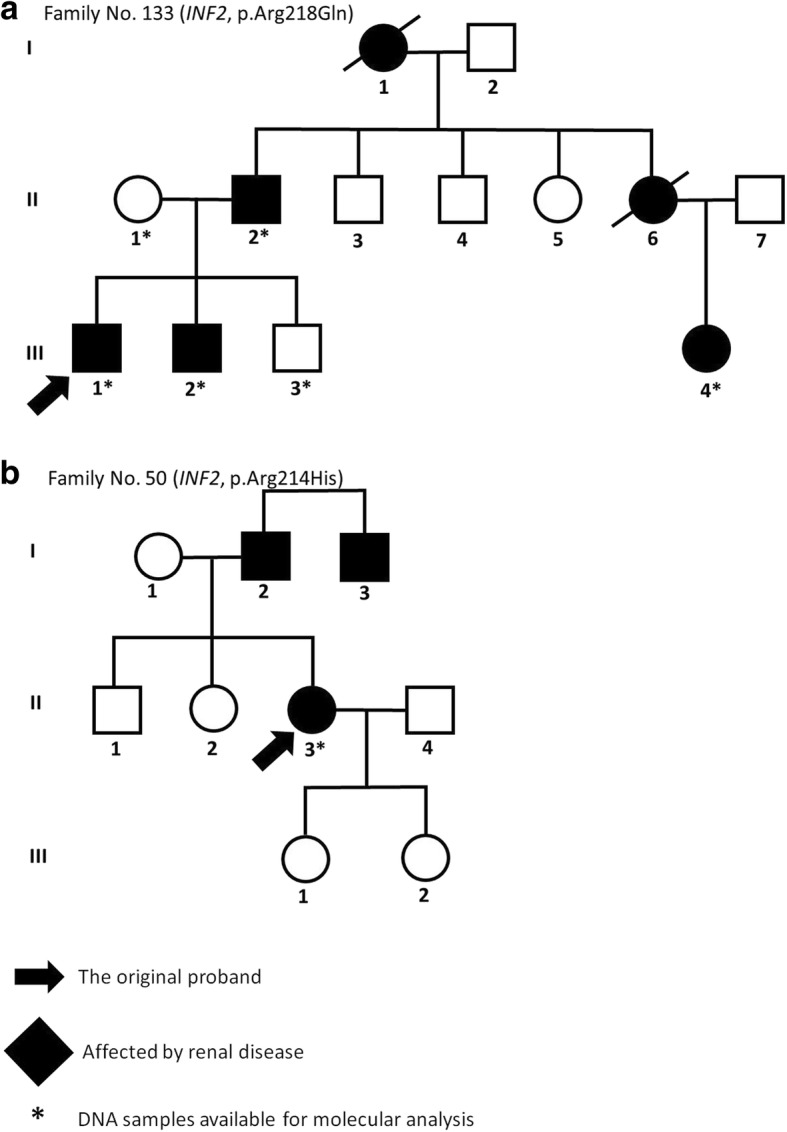
Pedigree diagrams of selected families from our cohorts of patients. The figure shows the pedigrees of the *INF2* mutated families; black arrows indicate original probands; black filled individuals were affected by renal disease. All affected individuals whose DNA was available for analysis carried the mutation in *INF2*

This family (see Fig. 1a), with a positive family history of ESRD of unknown etiology, and with mild proteinuria (affecting two brothers, their father, aunt, and cousin), had already been identified as one described by the causal missense mutation p.Arg218Gln (c.653 G > A) in *INF2*, with a damaging effect on podocyte function [5]. The substitution was first detected in the two brothers who had already developed ESRD. Afterwards, the same mutation was found in their father and cousin. The ages of the brothers at ESRD were 27 and 31 years, respectively; while their father developed ESRD at age 57. For that reason, in the case of these two patients, we presume the co-action of more factors, such as other substitutions in *INF2* or some influence from the environment. The younger affected brother as well as his healthy mother also had the SNP p.Pro528Ser (c.1582 C > T) in *INF2*. Additionally, we found other different intronic and exonic same-sense substitutions; however, there is not any hypothesis of their effects on the phenotype.

### Family no. 50 (*INF2*, p.Arg214His)

**Fig. 2:**
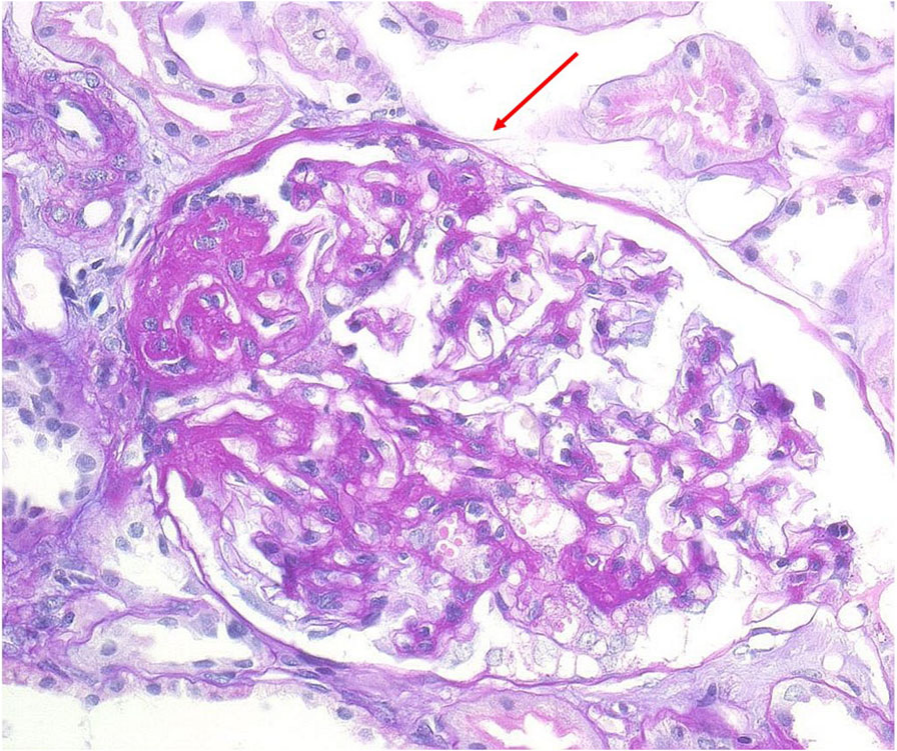
Kidney biopsy findings in a proband from family No. 50. Enlarged glomerulus with perihilar segmental sclerotic lesion. Simultaneously, there is showed a small sclerotic lesion (arrow) with a cell bridging between the Bowman capsule and the GBM (PAS, high power field, objective 40×)

Also identified was the known mutation p.Arg214His (c.641 G > A), with a proven damaging effect [5], in a 51 year old woman with FSGS (from a renal biopsy) and with a positive family history (her father had ESRD at age 33, and an uncle at age 30) (see Fig. 1b). Her two daughters were healthy. At her age, this patient has only mild chronic renal insufficiency with moderate proteinuria (1-3 g/24 h), although she suffers from diabetes mellitus, hypertension, and obesity (BMI = 35.58). The biopsy sample showed a perihilar variant of FSGS, with a mild increase of the mesangial matrix (see Fig. 2). This type of injury represents a common part of the so-called secondary FSGS, which is mediated by adaptive structural-functional responses, and frequently is associated with obesity of all of grades with a BMI **≥** 30.0. Even though the renal biopsy looked like secondary FSGS, a mutational analysis was done because of the positive family history of ESRD of unknown etiology.

## Discussion

Several genes highly expressed in podocytes (e.g., *ACTN4, INF2,* and *TRPC6*) had previously been reported to be a cause of familial and sporadic autosomal dominant forms of FSGS. Herein, we present the molecular genetic analysis of *INF2* (using the HRM method and Sanger sequencing) performed on a large cohort of patients with FSGS/MCD (FSGS = 77 patients, MCD = 32 patients), as well as in a group of patients (6) characterized by a positive family history for ESRD in combination with advanced CKD/ESRD at the time of diagnosis. These types of analyses can lead to the identification of new causal mutations; therefore, they are very important in clinical practice for the diagnosis of patients.

The INF2 protein, belonging to the formin family, plays a key role in the influence of the actin cytoskeleton dependent processes in podocytes because of its capability to accelerate both the polymerisation and depolymerisation of actin. The protein consists of three main parts including: the DID domain, two FH domains, and the DAD domain. Previous studies have demonstrated the abnormal distribution of the detected mutations; all of them having been identified in exons encoding the DID domain, suggesting its crucial role for the protein function. The DID domain appears to be important for actin cytoskeleton regulation [7, 8].

All identified substitutions in the exons were determined to be disease causing candidates if they: (a) segregated with the disease (in the case that other relatives were also affected, and we had samples of their DNA); (b) were predicted to be damaging by the Mutational Taster program [12], PolyPhen-2 [13], PROVEAN [14], and SIFT [15]; (c) were not carried by both healthy family members (whose DNA samples we had) as well as the healthy controls; and (d) were not present in control chromosomes in the db SNP and 1000 Genomes project.

We identified the mutation p.Arg218Gln in *INF2* with a damaging effect on the podocyte function in two brothers from a family with the positive history for ESRD. This mutation was first described by Brown et al. in a large family with the familial form of FSGS [5]. Both brothers quickly progressed to ESRD, aged 27 and 31, respectively.

The other detected casual mutation in *INF2* was p.Arg214His, also first identified by Brown et al. [5]. The mutation was found in a woman with a perihilar variant of FSGS and simultaneously suffering from diabetes mellitus. Although the family history of the proband was positive for renal diseases (father and uncle), her two healthy daughters had refused the opportunity for genetic testing. The incidences of this variant in our analysis were 0.9% (the whole cohort of patients), 1.3% (only the cohort of FSGS patients), and 5.6% (the cohort of FSGS patients with a positive family history), respectively. Both identified casual mutations are localized in the DID domain of INF2, which is necessary for normal protein function because it is involved in actin cytoskeleton regulation.

The mutational analysis of *INF2* also identified other different exonic changes and intronic substitutions that seemed to have no effect on the phenotype of the patients included in this study.

The most frequent substitution in *INF2* was p.P35P within exon 2, and with an allele frequency of more than 97%. This incidence corresponds with the results available in the ExAC Browser (http://exac.broadinstitute.org/) and gnomAD Browser (http://gnomad.broadinstitute.org/) databases, confirming the sensitivity of HRM. Other highly frequent changes in *INF2* were p.D1022D and p.D880D, with allele frequencies of 68% and 60%, respectively.

The HRM analysis was a very effective method for mutational screening in such a large cohort of patients. It proved to be of sufficient sensitivity in the detection of single nucleotide substitutions.

According to previous studies focused on *INF2*, it appears that this gene is responsible for a high percentage of patients with familial FSGS [5, 17, 18]. Even though the proportion of mutations in sporadic FSGS is significantly lower [9, 18], we included both familiar and idiopathic patients with FSGS. Although the incidence of pathogenic mutations was lower than our assumptions at the beginning of the study, we established the method for the molecular analysis of *INF2* and analyzed a large cohort of patients with FSGS/MCD; confirming that MCD cases are less likely to harbor deleterious genetic variants in those genes implicated in FSGS [4–6, 19]. This was the first molecular genetic analysis focused on *INF2* in the Czech Republic. A mutational analysis of *INF2* should be performed in all patients with a positive family history of FSGS or unknown ESRD with an autosomal dominant inheritance. Proteinuria is frequently mild or moderate, the patients are asymptomatic, and advanced renal insufficiency is already present, so a renal biopsy is not indicated in many cases.

## Conclusions

In conclusion, pathogenic mutations of *INF2* are not a frequent cause of FSGS nor of MCD in the Czech Republic. The *INF2* mutations must be taken into account; not only in patients with FSGS and a positive family history, but also in patients with ESRD of unknown etiology and a positive family history of ESRD in other generations. The HRM method seems to be a reliable and cheaper method than is direct sequencing for an analysis of the concrete gene in large cohorts of patients.

## Abbreviations

ACTN4: Alpha-actinin 4
BMI: Body mass index
CKD: Chronic kidney disease
DAD: Diaphanous autoregulatory domain
DID: Diaphanoury domain
ESRD: End stage renal disease
FH: Formin homology domain
FSGS: Focal segmental glomerulosclerosis
GBM: Glomerular basement membrane
HRM: High resolution melting
INF2: Inverted formin 2
MCD: Minimal change disease
PAS: Periodic acid-Schiff stain
R: Spectrin repeat
SNP: Single nucleotide polymorphism
TRPC6: Transient receptor potential cation channel, subfamily C, member 6.
